# Lateralised Behavioural Responses of Chickens to a Threatening Human and a Novel Environment Indicate Fearful Emotions

**DOI:** 10.3390/ani15142023

**Published:** 2025-07-09

**Authors:** Amira A. Goma, Clive J. C. Phillips

**Affiliations:** 1 Faculty of Veterinary Medicine, Alexandria University, Alexandria 21944, Egypt; 2 Curtin University Sustainability Policy Institute, Curtin University, Kent St., Bentley, WA 6102, Australia; clive.phillips@curtin.edu.au; 3 Institute of Veterinary Medicine and Animal Sciences, Estonian University of Life Sciences, Kreutzwaldi 1, 51006 Tartu, Estonia

**Keywords:** brain hemisphere, emotions, birds, human, novel, welfare

## Abstract

The demeanour of a human during an interaction with an animal may influence the animal’s emotional response. We investigated whether the emotional responses of laying hens to a threatening or neutral human and a novel environment were lateralised, from which their emotional state can be inferred. Twenty-five DeKalb white laying hens reared in furnished cages under environmentally controlled conditions were individually assessed for their responses to these stimuli. They were contained in a box before emerging into an arena with a threatening human, who attempted direct eye contact with the bird and had their hands raised towards it, or a neutral person, who had no eye contact and sat with their hands on their knees. When initially placed in the box adjacent to the test arena, birds that remained in the box used their left eye more than their right eye, and they showed evidence of nervousness, with many head changes, neck stretching, and vocalisation. Birds showed lateralised behaviour in both the box and arena. Birds entering the arena with the threatening person used their left eye (connected to the right brain hemisphere) more than their right eye, usually with their body less vertical, and were more likely to be standing than sitting, compared with those viewing the neutral person. This confirms the bird’s interpretation of the person as threatening, with left eye/right brain hemisphere processing of flight or fight situations. We conclude that lateralised responses of chickens suggest that a threatening person is viewed more fearfully than a neutral person. However, further investigation is required with a larger sample of birds to strengthen these findings and enhance the generalisability of behavioural responses.

## Simple Summary

It is hard to discern the emotional responses of an animal to a human being, but its behaviour is a key indicator. Chickens may detect a person’s demeanour and display fearful behaviour, including using their left eye, connected to their right brain hemisphere, which processes flight or fight reactions, more than their right eye to view the person. We compared the behaviour of chickens when confronted with a threatening person, who had their hands raised, reaching towards the bird with direct eye contact, with that of a neutral person, who sat with their hands on their knees and did not attempt to make eye contact. Birds viewing the threatening person used their left eye more to do this and had a more standing posture, compared with those viewing the neutral person. Birds also showed this lateralised behaviour more when first positioned in the test chamber. We concluded that chickens’ behavioural responses suggest that they are able to determine a person’s demeanour from subtle cues in their behaviour.

## 1. Introduction

The emotional state of animals is a critical component of animal welfare [1] and has featured more in welfare assessments recently [2]. Although their emotional state cannot be directly measured [3], it can be determined indirectly through behavioural and physiological assessments [4]. Emotional reactivity is the set of emotions experienced by animals that are processed in the brain to represent coping responses to changes [5]. One of the emotional states with a significant impact on an animal’s welfare is fear of human beings and novelty [5]. Fear is a response to perceived danger [6], usually a short, rapid response that motivates flight or freezing in reaction to a threat [7,8,9,10,11]. This helps the animal to avoid or cope with a threat through an adaptive behavioural pattern [12]. Although fear has a protective benefit, an exaggerated level of fear is considered a potent stressor that activates the hypothalamic–pituitary–adrenocortical axis, leading to negative consequences on animal health, welfare, and production [13,14,15,16]. In birds, fear is negatively related to exploratory behaviour [17,18]. An approach–avoidance conflict may be created when the animal is faced with a novel stimulus through stimulating exploratory behaviour together with avoidance behaviour to the threatening condition [19]. Avoidance suggests a fear reaction. When the animals’ ability to cope and adapt are exceeded, an imbalance between fear and exploration can lead to chronic stress [6].

Laying hens are typically housed in confined spaces under conventional intensive systems, restricting their ability to exhibit escape behaviours in response to novel objects or human presence [20,21]. Therefore, their fear reaction could be inappropriate and extremely damaging, as a response to their maladaptive environment. It includes panic, hysteria, and repeated escape attempts [20]. Fearfulness in birds can be measured by multiple tests, including the avoidance distance test, touch test, novel object test, and a stationary person test [22,23]. In the avoidance distance test, birds perceive human visual/physical contact as the most threatening if they are unaccustomed to human presence or have had unpleasant previous experiences with them [24].

It remains unclear how these responses are created and stored in the brain [25,26,27]. Chickens exhibit a complex behavioural repertoire. They possess sophisticated cognitive abilities, demonstrated by their exploration and learning performance in a Y-maze [28]. They also can orient themselves toward or relocate to a specific food resource in foraging contexts. [29]. Additionally, chickens display imprinting, passive avoidance learning, communication, self-control, mathematical abilities, and some concepts of physics [30]. Also, there is evidence of visual cognition and spatial orientation, recognition of occluded objects, time perception/anticipation, transitive inference, and discriminating among individuals [31]. They use their learning ability in exploratory, social, and antipredator behaviours [32]. Therefore, understanding the cognitive ability of chickens is important to their husbandry, production, and welfare [33,34,35].

Since emotional states take place in the brain [36], they are best indirectly assessed through behavioural and cognitive measurements and some physiological indicators [37]. Emotional reactivity and regulation can be predicted by cerebral lateralisation [38]. This approach is currently gaining interest and can be considered a non-invasive way to study the cerebral processing of emotions [36,39,40,41,42,43]. In cerebral lateralisation, each hemisphere is connected to the contralateral part of the body which it controls [44]. It can be detected through motor and sensory side biases. Therefore, behavioural observation can be an uncomplicated way to detect hemispheric specialisation in processing emotions, since asymmetrical behaviour can be indicated by lateralised motor responses under the control of the dominant brain hemisphere. Preferred limb use, head or tail turn, or complete body asymmetrical interactions with objects or conspecifics can be recorded and quantified [45]. This behavioural lateralisation indicates that the two hemispheres are different in their specialised processing of emotions [46].

Cozzutti and Vallortigara [47] studied the hemispheric memories for position of feed in chicks by exposing them to two different types of feed placed in two feeders positioned in different spatial locations. Chicks were eye-patched to create left-eye chicks (right eye patched), right-eye chicks (left eye patched), and binocular (unpatched) chicks. Binocular and right eye chicks succeeded in learning the position of feed, but not left eye chicks, demonstrating the role of lateralisation in learning and memory. Chickens have a complete optic chiasm decussation, in which each eye connects to the contralateral hemisphere [48,49].

Lateralised behaviour responses may be affected by exposure to light just before hatching; if the embryo’s posture occludes the left eye (LE) but not the right eye (RE), asymmetry in visual projections to the pallium is induced [50]. This exposure enhances the RE/left hemisphere’s ability to suppress attention to distracting visual cues and strengthens the left hemisphere’s inhibition of the right hemisphere, though it does not affect the right hemisphere’s interest in novelty. Light exposure before hatching has both immediate and long-term effects that are essential for species-typical behaviour and survival. For instance, in a food-search task under the threat of a predator overhead, dark-incubated chicks exhibited poor performance in both aspects of the task, whereas light-exposed chicks navigated it without difficulty. Additionally, steroid hormone levels prior to hatching regulate light-dependent asymmetry in visual projections, influencing neural competence for parallel processing and response inhibition [50].

The aim of the present study was to investigate the role of lateralisation in the emotional response of laying hens to different human approach conditions and a novel arena. Birds in controlled systems mostly do not experience a person at close range during rearing. At most, they will see a distant person checking the birds or providing feed. This is critical, as it significantly limits opportunities for direct human interaction beyond routine husbandry practices. This might heighten their fear-related lateralised responses when confronted with a threatening human or novel stimulus. Therefore, our hypothesis was that birds reared under controlled environmental conditions with little previous experience of human beings or novelty would express fear in their lateralised emotional responses (i.e., left eye/right hemisphere) in reaction to a threatening human or novelty.

## 2. Materials and Methods

### 2.1. Ethical Approval

The current study was conducted at the laying hen facility in the teaching and research poultry farm of Michigan State University, East Lansing, MI, USA from April to May 2023. The experimental design was approved by the IACUC committee of Michigan State University (PROTO202200159 and AMEND202300172).

### 2.2. Birds and Management

Three hundred and six *DeKalb white* laying hens were purchased from a commercial company and reared in accordance with the *Dekalb White* commercial management guide. They were housed in furnished cages (with pelleted feed, water drinkers, a perch, and a dust bath pad) in an environmentally controlled, windowless house at an age of 16 weeks. The birds were housed in six cages across two tier levels, with approximately 51 birds per cage. Diets were formulated to provide the recommended daily intakes of nutrients [51]. Water was supplied ad libitum. The temperature in the house was maintained within the thermal neutral zone (ETNZ) of 45 to 85 °F to within ±5 °F. The lighting provided was 5–10 lux for 11–16 h/d, varying as they aged.

### 2.3. Behavioural Assessment

At 25 weeks of age, 30 different hens (5 per cage) were randomly selected from various locations per cage for behavioural assessment using a familiarisation to the arena (*n* = 15) and human approach test (*n* = 15). Both tests were used for evaluation of the lateralisation of the behavioural responses of birds. Birds were video-recorded using a ceiling-mounted camera, providing an overhead view, and another on a tripod for a side view. Vocalisation was assessed by beak opening, as sound was heard, but was not recorded. However, during the testing, about 5 birds left the arena and ended the test, leaving a final sample of 25 hens—14 for familiarisation to the arena test and 11 for human approach test.

### 2.4. Arena and Adjacent Box

**Figure 1:**
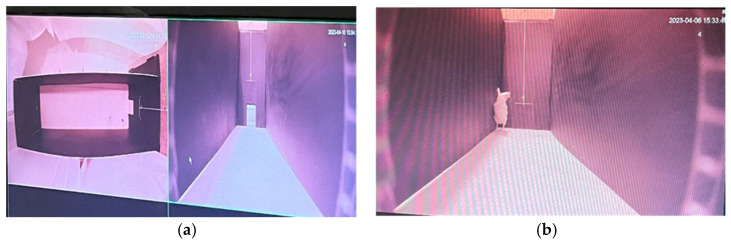
(**a**) Shape of arena from overhead and side views. (**b**) Side view of bird in arena with door closed.

The test arena (Figure 1) was rectangular (2.4 × 0.6 × 1.2 m), constructed from black corrugated polypropylene, with a box made from the same material attached to the door (19 × 40 × 30 cm) of the arena. The door between the two slid open to allow the birds entrance to the arena.

### 2.5. Familiarisation to the Arena Test

Each bird was tested once—they were transported individually by the researcher to the test room, which was adjacent to their home room, and placed in the box at the entrance to the arena. Four minutes were allowed for the bird to settle in the box before opening the door gently to allow them to view the arena for one minute. The emotional response of the bird to the box and arena environment was assessed each minute after opening the door, by one: zero sampling, using an ethogram, Table 1, compiled from various sources [6,52,53,54].

### 2.6. Human Condition Test

The end of the arena was opened to add a chair for a human (a different researcher from the one who transported the birds to the box) to sit on. A swinging door was inserted 0.8 m from the end of the arena, which was controllable from outside of the room to allow the bird to see the human. Before the start of the test, the human was seated on the chair wearing blue scrubs. The bird was transported to the test room, adjacent to the home room, and was placed at the entrance to the arena with the door closed. Three minutes were allowed for the bird to settle before opening the swinging door gently over the course of one minute.

**Figure 2:**
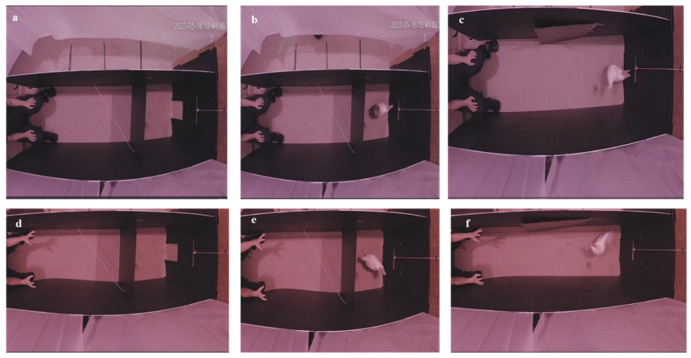
Neutral human (**a**) before bird entrance, and before (**b**) and after (**c**) opening the door. Threatening human (**d**) before bird entrance, and before (**e**) and after (**f**) opening the door.

There were two human conditions evaluated, neutral and threatening, with different birds randomly allocated to determine which they received. In the neutral condition, the human was sitting with their hands on their knees looking straight forward without interacting with the bird, while in the threatening condition the human was bending with their hands in the position of reaching towards the bird with direct eye contact, but without vocalising (Figure 2). The test lasted for 15 min after the swinging door opened, or until the bird left the arena. The mean recording duration was 8.27 min. The behaviour of the bird was continuously video-recorded and coded using the ethogram in Table 1 [6,52,54].

### 2.7. Statistical Analysis

After all video footage was coded by one: zero sampling for each minute of the test, the results were expressed as the frequency of each behaviour per minute of recorded bird video.

#### 2.7.1. Familiarisation to the Arena Test

##### Behaviour in the Box

The behaviour of birds that remained in the box was compared with that of post-emergence birds in the arena using GLM. Data residuals in both situations were assessed for normality by the Anderson Darling test using Minitab 19 and found to be not normally distributed (*p* < 0.005) for all behaviours. Logarithmic and square-root transformations were then performed, and residuals were tested again for normality by the Anderson Darling test using Minitab 19 and also found to be not normally distributed (*p* < 0.005) for all behaviours. Therefore, the Moods median non-parametric test was applied to determine the difference between the emotional responses of box birds and arena ones. The relationships between the emotional responses of birds were further explored by principal component analysis and stepwise regression analysis. The Kaiser–Meyer–Olkin measure of sampling adequacy to perform principal component analysis was determined, and it was found to be ~0.70, which is moderate adequacy for performing PCA. The association between twenty-two emotional responses (Appendix Table A1) was tested by principal component analysis (PCA). Six components had an eigen value > 1. Two components (5 and 6) were removed because of having no (6) or only one (5) variable with a coefficient more than 0.30 or the variable being present in other components with higher correlation coefficients.

##### Behaviour in the Arena

The Kaiser–Meyer–Olkin measure of sampling adequacy to perform principal component analysis was again determined, and it was found to be ~0.70, which is moderate adequacy for performing PCA. The association and relationship between emotional responses were therefore assessed by principal component analysis and stepwise regression analysis. In the principal component analysis, the association between twenty-eight emotional responses (Appendix Table A2) was evaluated. Nine components had an eigen value > 1. One component (8) was removed because it had no variable with a correlation coefficient more than 0.30 or the variable present in other components with higher coefficient, so there remained 8 components with 26 items representing 74.60% of total variance. After that, four components (2, 6, 7, and 9) were removed because they had only one variable with a correlation coefficient more than 0.30.

#### 2.7.2. Human Condition Test

The emotional responses of birds in the arena were found to be not normally distributed when tested by the Anderson Darling test using Minitab 19. Therefore, the Moods median test was used to determine the difference between the birds’ emotional responses to both human conditions.

## 3. Results

### 3.1. Familiarisation to the Arena Test

#### 3.1.1. Differences Between Birds’ Behaviours in the Box and the Arena

In the arena, birds tended to exhibit greater left-eye usage compared with right-eye usage, with an L+1/R+1 ratio of 1.25, though the difference in the ratio between the box and arena was not statistically significant (Table 2). However, birds used their left eye more in the arena than the box (*p* = 0.008), and they held their heads more down or level (*p* < 0.0001) than upwards. They were more likely to be sitting in the box and standing in the arena (*p* < 0.0001). Head turns, both to the left and right, were all more common in the arena than the box (*p* = 0.005; <0.0001 respectively). In the box, they were less likely to be in vertical position (*p* = 0.008), turning their body to the left (*p* < 0.0001) or holding up their left leg (*p* = 0.008) and pecking (*p* = 0.005) compared with their behaviour in the arena.

#### 3.1.2. Principal Components of the Birds’ Behaviour in the Box

**Figure 3:**
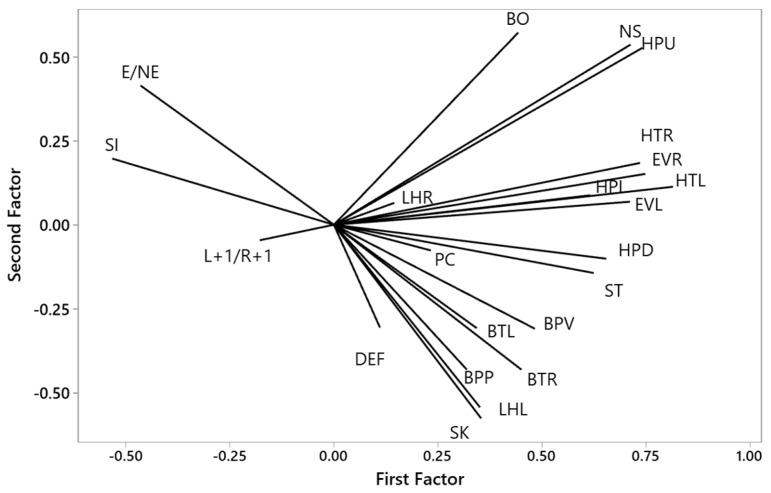
Loading coefficients > 0.30 for 19 lateralised emotional responses of laying hens within box in 4 components generated by principal component analysis. E/NE = emergence condition, E = emerged, NE = not emerged, L+1/R+1 = left eye view to right eye view ratio, EVR = eye view right, EVL = eye view left, BO = beak opening, NS = neck stretch, HPU = head position up, HPL = head position level, HPD = head position down, HTR = head turn right, HTL = head turn left, BTL = body turn left, BTR = body turn right, BPV = body position vertical, BPP = body position perpendicular, ST = standing, SI = sitting, DEF = defecation, LHL = leg held left, LHR = leg held right, PC = pecking, SK = shaking.

Four components with nineteen items representing 56.80% of total variance were retained. Component 1 consisted of most of the birds’ lateralised emotional responses: left eye view, right eye view, level head, head up, head down, neck stretch, head turn left, head turn right, body turn left, body turn right, body position vertical, body position perpendicular, and standing. The laterality of responses was therefore a key component of the birds’ responses to isolation within the box, pre-emergence. Component 2 consisted of emergence condition (whether the bird had emerged) and vocalisation. Shaking and left leg held were the main variables in component 3, and sitting and L+1/R+1 in component 4 (Figure 3, Appendix Table A1).

#### 3.1.3. Behaviours Connected with Left Eye View in the Box

Birds that used their left eye more than their right eye were more likely to have an outstretched neck, turn their head to the left, and withhold their right leg. They were less likely to vocalise, turn their head to the right, and be standing (Equation (1))

L+1/R+1 = 1.8574 (+0.0931) + 0.4102 (+0.0460, *p* < 0.0001) left eye view + 0.1436 (+0.0374, *p* < 0.0001) neck stretch—0.0997 (+0.0499, *p* = 0.048) vocalisation—0.1235 (+0.0524, *p* = 0.020) head turn right—0.2201 (+0.0911, *p* = 0.017) standing + 1.444 (+0.479, *p* = 0.003) right leg held—0.7078 (+0.0529, *p* < 0.0001) right eye view + 0.1488 (+0.0590, *p* = 0.013) head turn left (r^2^ = 84.24%). (1)

#### 3.1.4. Principal Components of Birds’ Behaviour in the Arena

**Figure 4:**
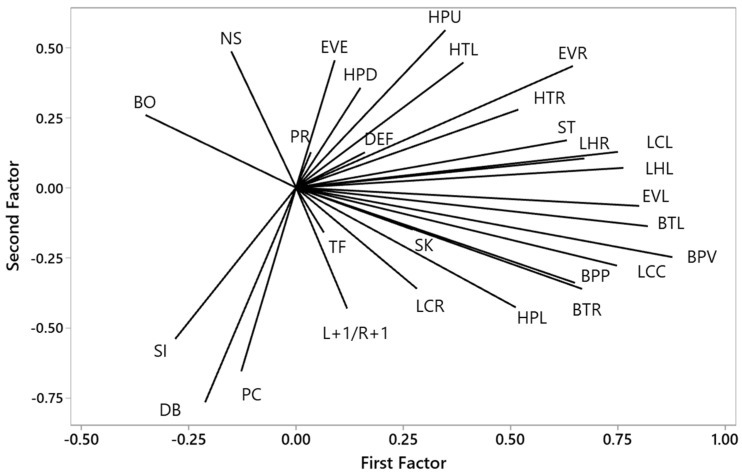
Loading coefficients > 0.30 for 23 lateralised emotional responses of laying hens within the arena in 4 components generated by principal component analysis. L+1/R+1 = left eye view to right eye view ratio, EVE = eye view emerge, EVR = eye view right, EVL = eye view left, BO = beak opening, NS = neck stretch, HPU = head position up, HPL = head position level, HPD = head position down, HTR = head turn right, HTL = head turn left, BTL = body turn left, BTR = body turn right, BPV = body position vertical, BPP = body position perpendicular, ST = standing, SI = sitting, DEF = defecation, LHL = leg held left, LHR = leg held right, PC = pecking, SK = shaking, PR = preening, TF = tail fanning, DB = dust bathing, LCL = location left, LCR = location right, LCC = location centre.

In the arena, there were four principal components (1, 3, 4, and 5) retained in the model, with twenty-three items representing 46.60% of total variance. Component one consisted of most of the birds’ lateralised emotional responses, which were left eye view, right eye view, level head, head turn right, body turn left, body turn right, body position vertical, body position perpendicular, left leg held, right leg held, location left, location centre, and standing (Figure 4, Appendix Table A2). Component 2 (3) consisted of head down, head turn left, pecking, and tail fanning. Neck stretch, vocalisation, and location right were the major behaviours in component 3 (4), and eye view during emergence and sitting in component 4 (5).

#### 3.1.5. Behaviours Connected with Left Eye View in the Arena

Birds that used their left eye more than their right eye to view the arena were less likely to have a level head, be sitting, and more likely to be dust-bathing (Equation (2))

L+1/R+1 = 1.073 (+0.130) + 0.2633 (+0.0265, *p* < 0.0001) left eye view—0.2105 (+0.0283, *p* < 0.0001) right eye view—0.0971 (+0.0419, *p* = 0.024) level head—0.336 (+0.128, *p* = 0.011) sitting + 0.1916 (+0.0361, *p* < 0.0001) dust bathing (r^2^ = 78.53%) (2)

### 3.2. Human Condition Test

L+1/R+1 was greater in the birds exposed to the threatening human, compared with the neutral human (*p* = 0.023), which was due to a combination of increased left eye view and decreased right eye view (Table 3). There was no difference in head or body orientation between the two treatments; however, there was a tendency (not significant) for birds presented with the threatening human to be more likely to be recorded in the horizontal position (*p* = 0.054) and less likely to be recorded in the vertical position (*p* = 0.036), compared with those viewing the neutral human. They were, however, more likely to be standing (*p* = 0.007) than sitting (*p* = 0.015). There was no difference between the two groups in location in the arena or in other behaviours.

## 4. Discussion

We investigated the role of lateralisation in the emotional response of laying hens individually assessed for their responses when familiarising themselves with the arena and when presented with two contrasting human demeanours. Birds in controlled systems mostly do not experience a person at close range during rearing, so we assume that the close presence of the person was novel to them. At most, they will see a distant person checking on them or providing feed. This significantly reduces opportunities for direct human interaction beyond routine husbandry practices. This might heighten their fear-related lateralised responses when confronted with a threatening human. Our hypothesis was that birds that had little previous experience with human beings reared under controlled environmental conditions express fear in their lateralised emotional responses (with an anticipated more use of the left eye/right hemisphere) in reaction to a threatening human.

In the familiarisation to the arena test, the birds used their left eye more than their right eye, with evidence of nervousness, many head changes, neck stretching, and vocalisation whilst sitting within the box. Birds showed evidence of lateralised behaviour in both the box and arena.

In the human condition test, birds entering the arena with the threatening person used their left eye (connected to the right brain hemisphere) more than their right eye, usually with their body less vertically, and were more likely to be standing than sitting, compared with those viewing the neutral person. This confirmed the hypothesis that the bird’s interpretation was that of a threatening person, recognising left eye/right brain hemisphere processing of flight or fight situations.

An animals’ emotional state is understood to be its internal, short-lived psychobiological responses to specific internal or external events which are biologically or ecologically relevant to the individual. These are conceptually distinct from moods, which are a more persistent internal state that is not necessarily triggered by a particular object or event [55]. Emotional state is accompanied by changes in the arousal and valence that can be determined by the alteration in behaviour, cognitive processing, physiology, and neural activity [55]. One of the animal’s emotional responses to the perceived danger is the fear response, displayed either physiologically or behaviourally. Although predators are not present in commercial poultry farms, there are still several aspects of poultry management that can provoke a fear response [56]. Furthermore, fearful birds are more challenging to manage and less capable of adapting to modifications in the environment, which can lead to management difficulties. Consequently, the ability to recognise individuals undergoing stress and having trouble in the maintenance of homeostasis can be specific bioindicators of a disrupted environment and welfare [57,58,59]. Birds’ fear can be assessed in numerous ways, and fear tests are becoming prevalent in poultry research.

Hemispheric specialisation is the distinct role of the left or right brain side in processing a specific neuronal task or behaviour [60]. One hemisphere may be predisposed to adopt a function due to specific structural and/or computational characteristics. This domination can be observed in enhanced sensory abilities, decision-making, and/or advanced motor abilities. Therefore, this hemisphere is responsible for organising and controlling motor responses that become visible as asymmetrical behaviour.

The previous literature on chicks has shown that each brain hemisphere processes information differently and controls different behaviours [61,62]. This side difference includes right hemisphere focus on novel and sudden events [63], sexual behaviour and aggression expression [64], attention to spatial relations between objects [65], social behaviour [66,67,68], and attention to the movement of living beings [69]. In particular, the crucial role of the right hemisphere in the creation of fear responses and the discovery of potential predators is well known [70]. On the other hand, the left hemisphere categorises stimuli dependent on past experience and learned behaviour [44,71]. In various mammalian species, the right hemisphere has been found to control social interactions, with a preference to keep a social partner on their left side [72].

Cerebral lateralisation is examined mainly from lateralised behaviours that indirectly mirror the activation of the right or left hemisphere [73]. Also, the assessment of the cognitive component of emotions includes the exploration of the processes regulating the emotional states, which are lateralised in both humans and animals [40,74]. The asymmetric management of different environmental stimuli through the varied senses (vision, olfaction, and audition) plus the lateralised motor patterns have been found to be correlated with different aspects of animal emotions [73]. The preference of using one nostril, ear, or eye to respond to a stimulus reflects the prevalent activity of the same-side hemisphere for olfaction [75] and contralateral hemisphere for vision and audition [76].

Chickens’ eyes are positioned on the sides of the head, with an angled head adopted for viewing objects monocularly if they are distant. Their binocular field of vision is used when viewing nearby objects (less than 30 cm in front of them) [77]. The preference for monocular vision when responding makes it practical to identify eye use and the contralateral hemisphere involved [78,79]. Furthermore, birds can shift their reliance between binocular and monocular vision by turning their head as they engage in a task, likely to optimise their perception and decision-making based on the specific demands of the situation. Such flexibility reflects the intricate neural coordination underlying their responses and the potential influence of lateralisation in guiding these shifts [80,81]. These insights not only deepen our understanding of avian behaviour but also suggest ways in which visual lateralisation could impact tasks like foraging or predator detection.

Moreover, lateralised motor patterns are indicated by the frequent use of a specific paw [82,83,84] or hand [85]. They can also be observed in the direction of movement of the entire body or a part of it [86,87,88,89,90,91]. These patterns emerge when an individual avoids an obstacle or explores a natural or laboratory test environment, such as a maze. Additionally, they manifest as a consistent turning bias during escape responses [60]. These reflect the activation of a specific brain hemisphere. The movements of extremities (e.g., limb/paw or tail) and the entire body are controlled by the motor cortex of the contralateral hemisphere [39]. Previous research has reported that these side biases in motor patterns are linked to various aspects of animal emotions, such as personality, temperament and coping abilities [92,93], vulnerability to stress, and behavioural style [39,94]. Therefore, lateralised motor behaviours, which are easy to measure, could provide information about how animals experience a specific condition that could impact on their well-being.

Generally, left limb lateralised individuals have been reported to show a high level of emotional reactivity and fear than right lateralised individuals, who appear to be more emotionally stable and proactive [91,93,95,96,97]. Lateralisation therefore provides critical information concerning individuals’ coping ability and vulnerability to stress [94]. Left limb/ambilateral individuals appeared to be more likely to adopt maladaptive behaviours in response to stressors, which significantly impacts their well-being [98]. Given that these lateralised motor behaviours are the demonstration of the cerebral functional asymmetries, it could be possible to gather information about individuals’ cognitive style by assessing their lateralised response in motor tasks [99].

Franklin and Lima [100] observed sparrows, noting how they position themselves to optimise their vigilance against predators by using the eye farthest from the wall. The preferred eye used to look outward was the left. The reaction of kookaburras is equally intriguing, as it suggests a left-eye system dominance possibly tied to spatially locating the prey on the ground while perching high on electrical wires [101].

Dharmaretnam and Rogers [102] deduced the ability of lateralised chicks to divide tasks between hemispheres—using the left hemisphere for finding grain and the right for responding to predators. The chicks switched rapidly between finding feed and vigilance for a predator instead of acting on both tasks at the same time. Also, head-down birds could distinguish an advancing overhead predator [103]. Therefore, they perform the two tasks in parallel, which signifies the advantage of lateralisation in multitasking and survival strategies. In contrast, non-lateralised chicks, which lack this division of labour, are at a disadvantage when facing these dual demands. This finding underscores the evolutionary benefits of lateralisation in enhancing cognitive and behavioural efficiency in birds.

A study on laying hens tested their ability to discriminate between three sounds which signed either positive (food reward), negative (a squirt from a water gun), or neutral (no sound) events after a 15 s delay [104]. The hens displayed varying emotional responses. In case of the negative sound, the hens exhibited more head movements than those experiencing neutral and positive sounds, which was related to anxiety. In the positive case, the birds presented comfort behaviours (preening, wing flapping, feather ruffling, and body scratching) that is consistent with relaxation [104].

Hemisphere specialisation and eye preferences in birds before performing a particular behavioural response is considered a practical indicator of the birds’ emotional state [40]. The left-eye system reveals emotional responses like fleeing or attacking, and the right-eye system denotes relaxation and controlled state. This understanding could be vital when assessing birds’ welfare under farming conditions, where environmental stressors might influence their emotional state and behaviour.

The notion that the left hemisphere dominance can enhance bird welfare by suppressing aggression and fear is a profound insight into how lateralisation affects emotional regulation in vertebrates. By inhibiting control by the right hemisphere, the left hemisphere likely promotes calmer and more adaptive behaviour in various situations, including interactions with their environment or other birds. This concept broadens our understanding of neural mechanisms not only in birds, but across vertebrate species [40,99]. It also underscores the potential applications of these findings in improving conditions for farmed birds and other animals in captivity, ensuring that environments that foster left-hemisphere-driven behaviours are fostered for better welfare outcomes.

A long period of research has shown a clear application of animal’s emotional state in animal welfare [105]. The association between the emotional response to an environment and the choice to avoid or approach that environment are fundamental elements in animal welfare [106]. Therefore, understanding farm animals’ emotions assists us to provide a better environment for both their welfare and human–animal relationships [107].

By observing head position and determining which eye is being used, researchers and caretakers can infer whether a bird is in a heightened state of alertness, experiencing stress, or engaging in calm, controlled behaviour. This insight allows for proactive measures to be taken, such as altering environmental conditions or minimising stressors to ensure the bird’s well-being. It also reflects the broader importance of lateralisation research in predicting behavioural responses, not just in birds, but across vertebrates. Such predictive capability can transform practices in farming, pet care, and conservation efforts.

We acknowledge that the small sample size of birds used in this study was a limitation. To strengthen these findings, future research should include a larger sample of birds to enhance the generalisability of behavioural responses. Expanding the study population would allow for a more robust assessment of individual variation and environmental influences on lateralised behavioural responses. We also acknowledge that vocalisations are not always associated with beak opening, and in the future, we will be using direct recordings of bird vocalisations.

## 5. Conclusions

In this study, we investigated whether the emotional responses of laying hens to a threatening or neutral human and a novel environment were lateralised, from which their emotional state can be inferred. Before emerging from their holding box into the arena, the birds used their left eye more than their right eye, and they showed evidence of nervousness, whilst sitting within the box. Birds entering the arena with a threatening person used their left eye (connected to the right brain hemisphere) more than their right eye, and were more likely to be standing than sitting compared with those viewing the neutral person. This confirms the bird’s interpretation of the person as threatening, with left eye/right brain hemisphere processing of flight or fight situations. We conclude that lateralised responses of chickens suggest that a threatening person is viewed more fearfully than a neutral person.

## Figures and Tables

**Table 1:** Ethogram of the birds lateralised emotional responses assessed in response to a novel arena and human.

Behaviour	Description
L+1/R+1	Ratio of left eye view (L) plus one to right eye view (R) plus one
Eye view left (EVL) *	Using left eye to view arena or human
Eye view right (EVR) *	Using right eye to view arena or human
Eye view before emerging (EVE) *^#^	Eye used to view arena just before emerging
Vocalisation (BO)	Opens the beak to give sound
Head position level (HPL)	Position of head in relation to the body
Head position up (HPU)	Position of head up in relation to the body
Head position down (HPD)	Position of head down in relation to the body
Head turn left (HTL) *	Turning head to left side
Head turn right (HTR) *	Turning head to right side
Body turn left (BTL) *	Turning whole body to the left side
Body turn right (BTR) *	Turning whole body to the right side
Body position vertical (BPV)	Position of body vertical in relation to floor
Body position perpendicular (BPP)	Position of body perpendicular in relation to floor
Neck stretch (NS)	Stretching neck with body stationary
Side of arena (location right = LCR) **^#^**	Bird present on right wall side of arena
Side of arena (location left = LCL) **^#^**	Birds present on left wall side of arena
Side of arena (location centre = LCC) **^#^**	The bird stands or passes in the centre of arena
Leg held left (LHL) *	Paused left leg when lifted before it is replaced again on ground or taking a step
Leg held right (LHR) *	Paused right leg when lifted before it is replaced again on ground or taking a step
Standing (ST)	Standing stationary without movement, includes head or body turn or other activities
Sitting (SI)	Sitting with bent legs underneath
Pecking (PC)	Pecking on ground or wall with beak
Shaking (SK)	Shaking body with ruffling of feathers
Defecation (DEF)	Defecation
Preening (PR)	Using a beak to groom self
Tail fanning (TF)^**#**^	Spreading tail feathers into a fan
Dustbathing (DB)^**#**^	Spreading wings, lowering body to ground and scratching ground or body with legs followed by shaking

* Lateralised behaviours are reported from the bird’s perspective. ^#^ Behaviours assessed in arena only.

**Table 2:** Impact of emergence condition (whether the bird had emerged into the arena or remained in the box) on behaviour of laying hens within box tested by Mood’s median test. Lateralised behaviours are reported from the bird’s perspective.

Variable *	Box vs. Arena	Median	*N* ≤ Median	*N* > Median	X^2^	*p*-Value
Left eye view	Box	2.00	68	31	7.14	0.008
	Arena	3.00	12	17		
Right eye view	Box	2.00	44	55	1.66	0.197
	Arena	2.00	9	20		
L+1/R+1	Box	1.00	51	48	0.40	0.526
	Arena	1.25	13	16		
Level head	Box	1.00	73	26	12.75	<0.0001
	Arena	2.00	11	18		
Head up	Box	0.00	56	43	0.02	0.894
	Arena	0.00	16	13		
Head down	Box	0.00	62	37	13.37	<0.0001
	Arena	2.00	7	22		
Neck stretch	Box	0.00	58	41	0.97	0.325
	Arena	1.00	14	15		
Vocalisation	Box	0.00	76	23	3.81	0.051
	Arena	0.00	27	2		
Head turn left	Box	1.00	69	30	7.74	0.005
	Arena	2.00	12	17		
Head turn right	Box	1.00	68	31	13.27	<0.0001
	Arena	2.00	9	20		
Body turn left	Box	0.00	96	3	13.88	<0.0001
	Arena	0.00	22	7		
Body turn right	Box	0.00	91	8	2.06	0.151
	Arena	0.00	24	5		
Body position vertical	Box	1.00	94	5	6.98	0.008
	Arena	1.00	23	6		
Body position perpendicular	Box	0.00	81	18	3.49	0.062
	Arena	0.00	19	10		
Standing	Box	0.00	54	45	27.36	<0.0001
	Arena	1.00	0	29		
Sitting	Box	1.00	31	68	38.42	<0.0001
	Arena	0.00	28	1		
Pecking	Box	0.00	93	6	8.03	0.005
	Arena	0.00	22	7		
Shaking	Box	0.00	98	1	3.40	0.065
	Arena	0.00	27	2		
Defecation	Box	0.00	98	1	3.40	0.065
	Arena	0.00	27	2		
Left leg held **	Box	0.00	99	0	6.94	0.008
	Arena	0.00	27	2		

* Values are expressed as the frequency of each behaviour per minute of recorded bird video. ** Right leg could not be calculated as there were too few observations.

**Table 3:** Impact of the human approach condition on lateralised emotional responses of laying hens evaluated by Mood’s median test. Represented by median, *N*≤, *N*> than median, chi-square value, and significance level.

Variable	Human Condition	Median	*N* ≤ Median	*N* > Median	X^2^	*p*-Value
L+1/R+1	Neutral	0.83	25	12	5.14	0.023
	Threatening	1.25	22	29		
Left eye view	Neutral	3.00	29	8	6.00	0.014
	Threatening	4.00	27	24		
Right eye view	Neutral	4.00	15	22	5.05	0.025
	Threatening	3.00	33	18		
Level head	Neutral	1.00	26	11	0.85	0.358
	Threatening	1.00	31	20		
Head up	Neutral	2.00	24	13	2.18	0.140
	Threatening	3.00	25	26		
Head down	Neutral	1.00	25	12	1.04	0.309
	Threatening	1.00	29	22		
Neck stretch	Neutral	1.00	21	16	0.13	0.723
	Threatening	1.00	27	24		
Vocalisation	Neutral	0.00	33	4	3.65	0.056
	Threatening	0.00	37	14		
Head turn left	Neutral	2.00	23	14	0.00	0.956
	Threatening	2.00	32	19		
Head turn right	Neutral	2.00	22	15	1.66	0.197
	Threatening	2.00	37	14		
Body turn left	Neutral	0.00	27	10	0.14	0.708
	Threatening	0.00	39	12		
Body turn right	Neutral	0.00	28	9	0.28	0.596
	Threatening	0.00	41	10		
Body position vertical	Neutral	1.00	8	29	4.42	0.036
	Threatening	1.00	22	29		
Body position perpendicular	Neutral	1.00	15	22	3.71	0.054
	Threatening	1.00	11	40		
Standing	Neutral	1.00	5	32	7.31	0.007
	Threatening	1.00	0	51		
Sitting	Neutral	0.00	31	6	5.95	0.015
	Threatening	0.00	50	1		
Left leg held	Neutral	0.00	31	6	0.03	0.860
	Threatening	0.00	42	9		
Right leg held	Neutral	0.00	27	10	0.14	0.708
	Threatening	0.00	39	12		
Location left	Neutral	0.00	25	12	1.90	0.168
	Threatening	0.00	27	24		
Location right	Neutral	1.00	16	21	1.59	0.207
	Threatening	0.00	29	22		
Location centre	Neutral	1.00	18	19	0.58	0.446
	Threatening	0.00	29	22		
Pecking	Neutral	0.00	29	8	0.00	0.995
	Threatening	0.00	40	11		
Shaking	Neutral	0.00	32	5	0.00	0.977
	Threatening	0.00	44	7		
Preening	Neutral	0.00	36	1	0.05	0.818
	Threatening	0.00	50	1		
Defecation	Neutral	0.00	32	5	1.51	0.219
	Threatening	0.00	48	3		
Dust bathing	Neutral	0.00	36	1	0.05	0.818
	Threatening	0.00	50	1		

## Data Availability

Please contact the primary author regarding data involved in this study.

## Appendix A

**Table A1:** Loading coefficients > 0.30 for 19 lateralised emotional responses of laying hens within the box in 4 components generated by principal component analysis. Lateralised behaviours are reported from the bird’s perspective.

Variable	Component 1	Component 2	Component 3	Component 4
Emergence condition		0.415		
Eye view left	0.712			
Eye view right	0.749			
L+1/R+1				0.664
Level head	0.617			
Head up	0.742			
Head down	0.655			
Neck stretch	0.713			
Vocalisation		0.573		
Head turn left	0.816			
Head turn right	0.737			
Body turn left	0.344			
Body turn right	0.451			
Body position vertical	0.483			
Body position perpendicular	0.320			
Left leg held			0.523	
Standing	0.625			
Sitting				0.402
Shaking			0.529	
Eigen value	6.260	2.519	1.958	1.767
Proportion (%) of r^2^	0.285 (28.5)	0.114 (11.4)	0.089 (8.9)	0.080 (8)

## Appendix B

**Table A2:** Loading coefficients > 0.30 for 23 lateralised emotional responses of laying hens within the arena in 4 components generated by principal component analysis.

Variable	Component 1	Component 3	Component 4	Component 5
Eye view emerge				0.560
Eye view left	0.800			
Eye view right	0.646			
Level head	0.512			
Head up		0.477		
Head down		0.510		
Neck stretch			0.739	
Vocalisation			0.749	
Head turn left		0.586		
Head turn right	0.518			
Body turn left	0.821			
Body turn right	0.667			
Body position vertical	0.878			
Body position perpendicular	0.650			
Left leg held	0.764			
Right leg held	0.673			
Location left	0.751			
Location right			0.492	
Location centre	0.749			
Standing	0.632			
Sitting				0.452
Pecking		0.439		
Tail fanning		0.480		
Eigen value	7.251	2.143	1.903	1.747
Proportion (%) of r^2^	0.259 (25.9)	0.077 (7.7)	0.068 (6.8)	0.062 (6.2)
